# Johns Hopkins Hospital Reports

**Published:** 1896-07

**Authors:** 


### Book Reviews.

---

Johns Hopkins Hospital Reports. Vol. V. Contents: I. Malarial Fevers of Baltimore; II. Study of Some Fatal Cases of Malaria; III. Studies in Typhoid Fever. Baltimore: The Johns Hopkins Press. 1895.

This report is divided into three parts—namely, 1. The malarial fevers of Baltimore; 2. A study of some fatal cases of malaria; 3. Studies in typhoid fever.

The first part shows careful study and wide investigation of the subject, as the table of references to the main works treating of malarial fever since the recognition of its parasitic origin indicates 359 authors consulted. It is also finely illustrated with three lithographs made in Leipzig. Ten divisions, besides the preliminary remarks, appear under the first subject and embrace the general knowledge of the malarial organism—namely, the analysis of 616 cases of malarial fever; varietiesof the hematozoa observed in malarial fevers of Baltimore; thegeneral analysis of 544 cases in which the type of organism was clearly distinguished; analysis of the types of fever associated with infection, with the different types of organism; concerning the nature and significance of the crescentic bodies of Laveran; the flagellate bodies; and the action of quinine on the malarial parasite; the relative susceptibility of the negro is by nearly two-thirds less than that of the white population; three varieties of malarial parasite are distinguished—1. The tertian; 2. The quartan; 3. The æstivo-autumnal.

Under part second, four fatal cases of malaria are reported, three of which are of the æstivo-autumnal and one of the double tertian type of malarial infection. Two chapters follow upon The unequal distribution of the parasites in the body in malarial infection, and Phagocytosis in malaria. This part is also illustrated with four fine lithographs.

The third and last part is full of interest to the general practitioner. Five out of the ten chapters of Studies in typhoid fever are from the pen of Dr. William Osler. With the general analysis given, and a summary of the cases, is a description of the special features, symptoms, complications, five years' experience with the cold bath treatment and corresponding low death-rate.

Pyuria as a complication of typhoid is discussed and mention made of ten cases, in seven of which the colon bacillus was present, in two the typhoid bacillus and in one the staphylococcus albus.

Certain forms of infection in typhoid fever, by Dr. Simon Flexner, is a valuable chapter. So-called lymphoid nodules of the liver in typhoid fever, Neuritis during and after typhoid fever and Chills in typhoid fever are interesting contributions. The last chapter is A study of the fatal cases : 1. Death by progressive asthenia ; 2. Death from intercurrent affections ; 3. Accidents of the lesion. This exhaustive report cannot fail to interest physicians everywhere. E. B. W.

